# Association between CT-Measured Abdominal Skeletal Muscle Mass and Pulmonary Function

**DOI:** 10.3390/jcm8050667

**Published:** 2019-05-12

**Authors:** Eun Kyung Choe, Young Lee, Hae Yeon Kang, Seung Ho Choi, Joo Sung Kim

**Affiliations:** 1 Department of Surgery, Healthcare Research Institute, Seoul National University Hospital Healthcare System Gangnam Center, Seoul 06236, Korea; choe523@gmail.com; 2 Veterans Medical Research Institute, Veterans Health Service Medical Center, Seoul 05368, Korea; lyou7688@gmail.com; 3 Department of Internal Medicine, Healthcare Research Institute, Seoul National University Hospital, Healthcare System Gangnam Center, 737 Yeoksam-dong, Gangnam-gu, Seoul 06236, Korea; cshmed@snuh.org (S.H.C.); jooskim@snu.ac.kr (J.S.K.); 4 Department of Internal Medicine, Liver Research Institute, Seoul National University College of Medicine, Seoul 03080, Korea

**Keywords:** lung function, skeletal muscle, sarcopenia, CT

## Abstract

A relationship between lung function and sarcopenia has been suggested. This study aimed to evaluate the association between lung function and abdominal skeletal muscle mass, as measured by computed tomography (CT). The clinical records of 1907 subjects (1406 males, mean age 53.1 ± 9.2 years), who underwent routine health check-ups, including spirometry and abdominal CT, were retrospectively reviewed. The CT-measured skeletal muscle index (SMI_CT_, cm^2^/(kg/m^2^) was defined as the skeletal muscle area of the third lumbar vertebrae (L3) level that is normalized by the body mass index. The mean values of forced vital capacity (FVC) and forced expiratory volume in one second (FEV1) gradually increased as the SMI_CT_ quartiles increased (all *p* for trend < 0.05). The proportions of subjects with less than 80% of the predicted FVC (%) and predicted FEV1 (%) significantly decreased as the SMI_CT_ quartiles increased (all *p* for trend < 0.05). The β regression coefficients for FVC and FEV1 significantly increased as the SMI_CT_ quartiles increased after adjusting for other confounding variables (*p* for trend < 0.05). This study showed that abdominal muscle mass, which was precisely measured by CT, independently affected lung function proportionally after adjusting for confounding factors in relatively healthy adults.

## 1. Introduction

Sarcopenia, which is described as the progressive loss of skeletal muscle mass and strength associated with aging [1,2], is related to numerous health problems, such as cardiovascular disease, fatty liver disease, metabolic disorders, and risk of death in elderly individuals [3,4,5,6]. Sarcopenia is reportedly associated with the lung function, quality of life, and survival of people with chronic obstructive pulmonary disease (COPD) and lung cancer [7,8,9,10]. However, the pathophysiology of sarcopenia in people with lung disease is different from that in relatively healthy people.

Recent studies have reported that low skeletal muscle mass is associated with poor lung function in asymptomatic subjects without clinically apparent lung disease [11,12,13]. A study on the relationship between skeletal muscle mass and lung function in relatively healthy adults used generalized skeletal muscle mass, as measured by bioelectrical impedance analysis (BIA) [13]. Another recent study revealed that poor pulmonary function was associated with low muscle mass, as determined by dual-energy X-ray absorptiometry (DEXA), in Korean subjects that were over 65 years old [11]. The European consensus defined computed tomography (CT) scans and magnetic resonance imaging (MRI) as the gold standards in estimating muscle mass [2], and skeletal muscle area can be objectively measured on cross-sectional images as a valid surrogate for whole-body muscle mass [14,15,16]. In this study, the abdominal skeletal muscle mass of the third lumbar vertebrae (L3) level was accurately measured while using CT.

We aimed to investigate the relationship between lung function, as measured by forced vital capacity (FVC), and forced expiratory volume in one second (FEV1) using spirometry, and abdominal skeletal muscle mass, as measured by CT, in healthy subjects.

## 2. Materials and Methods

### 2.1. Study Subjects

We performed a cross-sectional study to evaluate the association between CT-measured skeletal muscle mass and lung function. The clinical records of 2106 subjects who underwent routine health check-ups, which included spirometry and abdominal fat CT, between January 2009 and December 2014 at the Seoul National University Hospital Healthcare System Gangnam Center, were reviewed. We excluded 166 subjects who were under 18 years of age, did not complete the questionnaire, or did not know the required clinical information, such as their body mass index (BMI). Additionally, 33 subjects who had a history of cancer, chronic obstructive pulmonary lung diseases, or tuberculosis were excluded. Finally, 1907 participants were enrolled in this study.

### 2.2. Ethics Statement

The Institutional Review Board of Seoul National University Hospital approved this study protocol (H-1606-095-771) and waived the requirement for informed consent. The study was performed in accordance with the Declaration of Helsinki.

### 2.3. Measurements and Definitions

All of the participants answered a questionnaire regarding their medical history and completed an anthropometric assessment and laboratory test on the same day. The participants were grouped as never, ex-, or current smokers and, as no regular exercise, moderate activity, or rigorous activity (≥3/week) based on a self-reported medical questionnaire. Systolic and diastolic blood pressure (BP) were measured twice and the mean values were recorded. A well-trained nurse measured waist circumference (WC) at the midpoint between the lower costal margin and the iliac crest. The BMI (kg/m^2^) was calculated as the weight divided by the height squared. All of the blood samples were collected after the participants had fasted for at least 12 h. The laboratory evaluation included total cholesterol (TC), triglyceride (TG), low-density lipoprotein (LDL) cholesterol, high-density lipoprotein (HDL) cholesterol, fasting glucose, and hemoglobin A1c (HbA1c) levels.

The skeletal muscle area was measured, as described in previous studies [4,17]. The participants were examined with a 16-detector row CT scanner (Somatom Sensation 16; Siemens Medical Solutions, Forchheim, Germany). L3 was selected as the standard landmark; the L3 region contains the psoas, paraspinal, and abdominal wall muscles. We used a CT software program (Rapidia 2.8; INFINITT, Seoul, Korea) to electronically determine the skeletal muscle area by setting the attenuation values for a region of interest within a range of −29 to 150 Hounsfield units, as previously described [4,14]. A trained technician corrected the boundary of the entire L3 skeletal muscle area twice, and the average value was used for analysis. This value was adjusted by the BMI (kg/m^2^), according to the guidelines of the Foundation for the National Institutes of Health (NIH) Sarcopenia Project [15], and reported as the CT-measured skeletal muscle index (SMI_CT_, cm^2^/(kg/m^2^) [4]. The study participants were stratified by sex and divided into four groups according to SMI_CT_ quartiles; the cutoff quartile values for males were 8.53, 9.29, and 10.15 cm^2^/(kg/m^2^), while those for females were 7.26, 7.94, and 8.94 cm^2^/(kg/m^2^).

Experienced technicians performed spirometric tests according to American Thoracic Society recommendations [18], and a dry rolling seal spirometer was used (model 2130, Viasys Respiratory Care, Inc., San Diego, CA, USA). The FVC, FEV1, and FEV1/FVC were measured and they are expressed as both absolute values (liters) and predicted values (%), as calculated from the formula based on the Korean population [19,20]. The following cutoff values were used to represent pulmonary dysfunction, as measured by a pulmonary function test (PFT): FVC (%) < 80%, FEV1 (%) < 80%, and FEV1/FVC < 70% of the predicted values [21].

### 2.4. Statistical Analyses

All data are presented as the mean ± standard deviation or number (percentage) based on the SMI_CT_ quartiles. The demographic characteristics and PFT results were compared while using the chi-squared test or one-way analysis of variance (ANOVA) among the four groups. We hypothesized that the PFT parameter values would gradually increase as the SMI_CT_ quartiles increased. Linear regression was used to assess the relationship between SMI_CT_ and PFT parameters. As no standard values are available for defining a normal abdominal muscle mass quantity, we divided the SMI_CT_ into four groups (cutoff values in quartiles (cm^2^/(kg/m^2^)): for males, 8.53, 9.29, 10.15; for females, 7.26, 7.94, 8.94). All of the statistical analyses were separately performed according to sex, because skeletal muscle values, pulmonary function values, and other independent variables are significantly different between males and females. The association between abdominal muscle mass and lung function was evaluated while using multiple linear regression models, and the β regression coefficients for lung function according to the SMI_CT_ quartiles are presented. Logistic regression was used to assess the association of the SMI_CT_ and PFT parameters with a binary outcome, and the results are presented as odds ratios (ORs) with 95% confidence intervals (CIs). The variables that are known risk factors and were statistically significant in the univariate analysis were included in a multiple regression model to identify the independent predictors of lung function and sarcopenia. Multicollinearity was assessed while using the variance inflation factor (VIF). The *p* value for trend was calculated by comparing the model with the quartile group variable to that without the quartile group variable.

Statistical analyses were performed using Statistical Package for the Social Sciences software, version 22.0 (SPSS, Inc., Chicago, IL, USA) and R statistical software, version 3.2.2 (R Development Core Team; R Foundation for Statistical Computing, Vienna, Austria). Statistical significance was established for two-sided *p* values < 0.05.

## 3. Results

Table 1 describes the characteristics of 1907 participants according to SMI_CT_ quartiles (Tables S1 and S2 according to sex). The proportion of male participants was 73.7% and the mean age was 53.1 ± 9.2 years. The mean SMI_CT_ value for the participants was 9.1 ± 1.4 (9.4 ± 1.3 in males and 8.2 ± 1.4 in females). Age, systolic BP, diastolic BP, weight, BMI, WC, TC, triglycerides, LDL cholesterol, fasting glucose, and HbA1c values significantly decreased as the SMI_CT_ quartiles increased. However, height and HDL cholesterol values significantly increased as the SMI_CT_ quartiles increased. There were more never smoking participants and fewer current smoking participants in the group with low SMI_CT_.

**Figure 1:**
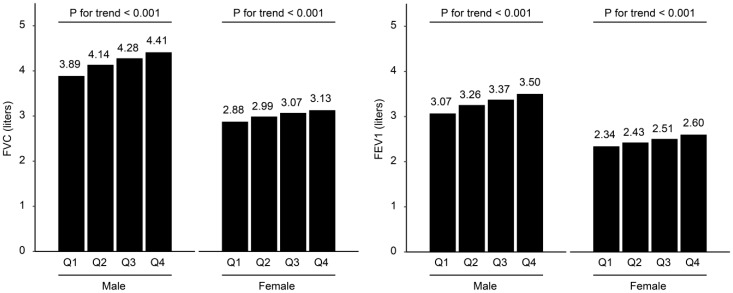
Lung function according to the computed tomography (CT)-measured skeletal muscle index (SMI_CT_) quartiles in males and females. Cutoff values of SMI_CT_ quartiles (cm^2^/(kg/m^2^)) for males, 8.53, 9.29, 10.15; and for females, 7.26, 7.94, 8.94.

Table 2 presents the mean PFT parameter values and the proportions of participants with low PFT values, according to the SMI_CT_ quartiles (Tables S3 and S4 according to sex). The mean values of FVC (liters), predicted FVC (%), FEV1 (liters), and predicted FEV1 (%) gradually increased as the SMI_CT_ quartiles increased (all *p* for trend < 0.05). The proportions of participants with less than 80% of the predicted FVC (%) and predicted FEV1 (%) significantly decreased as the SMI_CT_ quartiles increased (all *p* for trend < 0.05). However, FEV1/FVC (%) and the proportion of participants with FEV1/FVC < 70% were not significantly associated with the SMI_CT_ quartiles. Figure 1 shows the mean values of FVC (liters) and FEV1 (liters), according to the SMI_CT_ quartiles in males and females. The mean values of FVC (liters) and FEV1 (liters) gradually increased as the muscle mass increased in males and females.

Table 3 shows the association between abdominal muscle mass and lung function. In males and females, SMI_CT_ was associated with both the FVC and FEV1. The β regression coefficient for each predictor for SMI_CT_ values remained significant, except for FVC in females after adjusting for age, BMI, smoking, exercise, systolic BP, diastolic BP, height, cholesterol, TG, HDL cholesterol, and HbA1c. However, SMI_CT_ was not associated with the FEV1/FVC ratio.

Table 4 presents the β regression coefficients for lung function according to SMI_CT_ quartiles. After adjusting for age, BMI, smoking, exercise, systolic BP, diastolic BP, height, cholesterol, TG, HDL cholesterol and HbA1c, the β regression coefficients for FVC and FEV1, except FEV1 for females, significantly increased as the SMI_CT_ quartiles increased (*p* for trend < 0.05 in males and females).

As shown in Table 5, multiple logistic regression analyses adjusted for age, BMI, smoking, exercise, systolic BP, diastolic BP, height, cholesterol, TG, HDL cholesterol, and HbA1c were performed for SMI_CT_ quartiles and subjects with low PFT values. The adjusted ORs for participants with an FVC (%) < 80% for Q1, Q2, and Q3 when compared with Q4 (reference) were 3.52 (1.75, 7.10), 2.00 (0.98, 4.05), and 1.94 (1.00, 3.79), respectively (*p* for trend <0.005). Adjusted ORs for participants with an FEV1 (%) < 80% for Q1, Q2, and Q3, as compared with Q4, had trends that are similar to FVC (%), but the trends were not significant (2.55 (1.03, 6.31), 2.18 (0.91, 5.23), and 1.84 (0.77, 4.37), respectively, *p* for trend > 0.05).

## 4. Discussion

In this study, participants with low muscle mass had low FVC or FEV1 values, and lung function proportionally increased according to the SMI_CT_ quartile. These relations persisted after adjustment for various confounding factors. When compared to that in the highest SMI_CT_ quartile group, the lowest SMI_CT_ quartile group had more than twice the number of participants with low FVC or FEV1 values. These results showed that abdominal muscle mass proportionally affected lung function, even if the muscle mass was not reduced to a sarcopenic level in a relatively healthy adult. This result was not consistent with those of a previous study, in which an exercise program increased muscle mass in all of the participants, but increased lung function in only participants with sarcopenia [22]. However, that study included only a small number of participants, and appendicular skeletal muscle mass was measured with DEXA, which was unlike our study.

In the present study, FEV1/FVC was not associated with skeletal muscle mass, unlike FEV1 and FVC, and these findings are consistent with those of previous studies [12,13]. FVC usually represents lung volume, and FEV1 represents the expiration flow rate; therefore, FVC and FEV1 can be reduced in participants with low muscle mass, because they may have a weakened ability to inflate and deflate their lungs. However, FEV1/FVC represents upper airway obstruction and may remain invariant, regardless of muscle mass [13,18].

When we performed analyses that were stratified by sex, males had more significant results than females. Some nonsignificant results after the correction of confounding factors in females seemed to be due to the small number of females in this study. These results are consistent with those of a previous study that found that adjusted ORs for <80% of FVC and FEV1 were higher in males than females [13]. During the aging process, the decrease of FEV1 and FVC with age is greater in males than females [23]. Previous reports explain that oxidative stress is more evident in males, which might influence lung function as muscle mass decreases [13,24]. In addition, other possible explanations for sex differences have been suggested, which include differences in sex hormone levels, thresholds for the harmful effects of pulmonary irritants, and differences in the airway caliber between males and females [25,26,27]. However, more research on the effects of sex on skeletal muscle mass and lung function is needed.

A possible mechanism that links low skeletal muscle mass and pulmonary dysfunction has been suggested. The interaction among the lungs, chest wall, and respiratory muscles influence respiratory function [23,28]. Systemic inflammation driven by cytokines and oxidative stress has been suggested as a mechanism underlying skeletal muscle mass loss [29,30,31]. Chronic, low-grade inflammation could lead to muscle proteolysis and myocyte apoptosis [29,32]. In addition, the high-sensitivity C-reactive protein concentration was negatively associated with lung function or muscle mass [33]. Physical activity plays an important role in maintaining respiratory muscle strength [34,35]. Muscle loss with low physical activity leads to further muscle loss and it induces cyclic pulmonary dysfunction.

Furthermore, the relationship between poor lung function and skeletal muscle mass could be explained by sarcopenia of the respiratory muscles themselves, such as the diaphragm, which has been demonstrated in aging mice [36]. Respiratory skeletal muscle is known to be related to generalized skeletal muscle mass and it undergoes the same sarcopenic process as other skeletal muscles [23,28,36,37]. Previous studies suggest that respiratory muscle strength and peripheral muscle strength are interrelated, and that sarcopenia may affect respiratory muscle strength [38,39]. We used the cross-sectional skeletal muscle at the L3 level, as this value is known to be linearly related to whole-body muscle mass [4,40]. Our results suggest that abdominal skeletal muscle mass can affect lung function and indirectly represent respiratory skeletal muscle mass. Further research regarding the mechanism underlying the relationship between skeletal muscle mass and lung function in relatively healthy subjects is warranted.

The present study has several strengths. First, this study only included relatively healthy individuals without known lung disease, and many potential confounders were considered in the questionnaires and labs. Second, abdominal skeletal muscle mass was precisely measured by CT. BIA and DEXA also reflect muscle mass well, but CT and MRI are considered as the gold standards for muscle mass measurement [2]. While the previous study published by Park et al. [13] was large, the authors used BIA, which is a less reliable method for quantifying body composition than CT in terms of accuracy, to observe the relationship between whole-body muscle and lung function [6,13]. Third, we demonstrated that lung function proportionally increased with abdominal skeletal muscle mass. Therefore, our results suggest that increasing abdominal skeletal muscle mass will help to improve lung function in relatively healthy subjects.

However, this study does have several limitations. First, this study was cross-sectional and retrospective in nature, which thereby makes it difficult to clarify the temporal or causal relationship between skeletal muscle mass and lung function. Second, the participants in this study were self-recruited from a single center during routine health check-ups; therefore, the socioeconomic statuses of the study participants were relatively good, and the participants may have been more interested in health. As such, our findings might not represent the general population. Third, we did not have data on muscle function. We could better understand the correlation between muscle and lung function if we considered muscle strength or performance in addition to muscle mass.

In conclusion, this study showed that abdominal skeletal muscle mass, which was precisely measured by CT, independently affected lung function proportionally after adjusting for confounding factors in relatively healthy adults. Therefore, improved lung function can be expected when abdominal skeletal muscle mass is increased. For subjects with low abdominal skeletal muscle mass, lung function may linearly decrease; therefore, more muscle strength training should be recommended. In the future, a well-designed prospective, longitudinal study is needed to evaluate the association between changes in lung function and changes in abdominal skeletal muscle mass.

## Figures and Tables

**Table 1:** Comparison of the baseline characteristics of participants according to CT-measured skeletal muscle index (SMI_CT_) quartiles.

Variable	Total (*n* = 1907)	SMI_CT_ Quartiles				***p* for Trend**
		Q1 (*n* = 478)	Q2 (*n* = 476)	Q3 (*n* = 476)	Q4 (*n* = 477)	
Age, years	53.1 ± 9.2	55.7 ± 8.9	53.5 ± 9.0	52.2 ± 8.5	51.1 ± 9.6	<0.001
Male (%)	1406 (73.7%)	352 (73.6%)	351 (73.7%)	351 (73.7%)	352 (73.8%)	0.959
Systolic BP, mmHg	117.3 ± 13.7	119.9 ± 13.3	118.7 ± 13.4	116.3 ± 13.7	114.3 ± 13.8	<0.001
Diastolic BP, mmHg	76.2 ± 10.8	77.4 ± 10.5	77.7 ± 10.9	75.5 ± 10.8	74.4 ± 10.8	0.001
Height, cm	167.2 ± 7.4	164.9 ± 7.2	166.7 ± 7.3	167.9 ± 7.2	169.2 ± 7.4	<0.001
Weight, kg	66.8 ± 10.9	68.7 ± 10.5	67.9 ± 11.0	66.5 ± 10.6	64.1 ± 11.0	<0.001
BMI, kg/m^2^	23.8 ± 2.8	25.1 ± 2.7	24.3 ± 2.5	23.5 ± 2.5	22.3 ± 2.6	<0.001
WC, cm	85.7 ± 7.8	89.1 ± 7.2	87.0 ± 7.4	85.1 ± 7.1	81.6 ± 7.7	<0.001
Smoking						
Never	779 (40.8%)	216 (45.2%)	202 (42.4%)	179 (37.6%)	182 (38.2%)	0.010
Ex-smoker	769 (40.3%)	197 (41.2%)	199 (41.8%)	191 (40.1%)	182 (38.2%)	0.280
Current smoker	359 (18.8%)	65 (13.6%)	75 (15.8%)	106 (22.3%)	113 (23.7%)	<0.001
Exercise						
No regular exercise	548 (28.7%)	157 (32.8%)	135 (28.4%)	124 (26.1%)	132 (27.7%)	0.054
Moderate activity	654 (34.3%)	146 (30.5%)	156 (32.8%)	188 (39.5%)	164 (34.4%)	0.061
Rigorous activity (≥3/week)	705 (37.0%)	175 (36.6%)	185 (38.9%)	164 (34.5%)	181 (37.9%)	0.968
Muscle, cm^2^	215.2 ± 36.4	191.7 ± 29.7	209.0 ± 30.3	220.0 ± 32.2	240.2 ± 35.2	<0.001
SMI_CT,_ cm^2^/(kg/m^2^)	9.1 ± 1.4	7.6 ± 0.7	8.6 ± 0.6	9.4 ± 0.6	10.8 ± 1.0	<0.001
TC, mg/dL	192.1 ± 33.9	195.7 ± 35.1	196.0 ± 37.2	188.6 ± 31.6	188.2 ± 30.6	<0.001
TG, mg/dL	121.6 ± 78.8	132.2 ± 82.8	134.0 ± 89.0	118.4 ± 75.7	101.7 ± 60.7	<0.001
HDL cholesterol, mg/dL	52.9 ± 12.8	51.3 ± 11.9	51.9 ± 12.2	52.5 ± 12.8	55.9 ± 13.7	<0.001
LDL cholesterol, mg/dL	119.7 ± 29.5	125.1 ± 30.8	121.3 ± 30.9	117.0 ± 29.1	115.9 ± 26.5	<0.001
Fasting glucose, mg/dL	98.8 ± 16.6	100.2 ± 15.5	99.9 ± 16.0	98.3 ± 17.2	96.6 ± 17.4	<0.001
HbA1c, %	5.8 ± 0.5	5.8 ± 0.5	5.8 ± 0.5	5.7 ± 0.5	5.7 ± 0.5	<0.001

Data are presented as the mean ± standard deviation or as a percentage (%). Cutoff values of SMI_CT_ quartiles (cm^2^/(kg/m^2^)) for males, 8.53, 9.29, 10.15; and for females, 7.26, 7.94, 8.94. Q: quartile; BP: blood pressure; BMI: body mass index; WC: waist circumference; TC: total cholesterol; TG: triglyceride; LDL: low-density lipoprotein; HDL: high-density lipoprotein; HbA1c: hemoglobin A1c.

**Table 2:** Comparison of PFT values between participants according to CT-measured skeletal muscle index (SMI_CT_) quartiles.

Variable	Total (*n* = 1907)	SMI_CT_ quartiles				***p* for Trend**
		Q1 (*n* = 478)	Q2 (*n* = 476)	Q3 (*n* = 476)	Q4 (*n* = 477)	
FVC, liters	3.9 ± 0.8	3.6 ± 0.7	3.8 ± 0.7	4.0 ± 0.8	4.1 ± 0.8	<0.001
FVC, % predicted	96.8 ± 11.5	95.0 ± 11.4	96.7 ± 11.3	97.3 ± 11.9	97.9 ± 11.2	<0.001
Predicted FVC(%) < 80%	101 (5.3%)	35 (7.3%)	24 (5.0%)	25 (5.3%)	17 (3.6%)	0.016
FEV1, liters	3.1 ± 0.7	2.9 ± 0.6	3.0 ± 0.6	3.1 ± 0.6	3.3 ± 0.7	<0.001
FEV1, % predicted	104.2 ± 13.7	102.9 ± 14.0	103.7 ± 13.5	104.4 ± 14.2	105.7 ± 13.0	0.002
Predicted FEV1 (%) < 80%	64 (3.4%)	21 (4.4%)	19 (4.0%)	15 (3.2%)	9 (1.9%)	0.023
FEV1/FVC (%)	79.7 ± 6.5	79.5 ± 6.4	79.3 ± 6.1	79.5 ± 6.5	80.3 ± 6.8	0.061
FEV1/FVC < 70%	105 (5.5%)	28 (5.9%)	24 (5.0%)	28 (5.9%)	25 (5.2%)	0.828

Data are presented as the mean ± standard deviation or as a percentage (%). Cutoff values of SMI_CT_ quartiles (cm^2^/(kg/m^2^)) for males, 8.53, 9.29, 10.15; and for females, 7.26, 7.94, 8.94.

**Table 3:** Association between CT-measured skeletal muscle index (SMI_CT_) and lung function.

Variable	FVC			FEV1			FEV1/FVC (%)		
	β	SE	*p* Value	β	SE	*p* Value	β	SE	***p* Value**
Male									
SMI_CT_, cm^2^/(kg/m^2^) *^1^*	0.132	0.013	<0.001	0.094	0.011	<0.001	−0.252	0.140	0.071
SMI_CT_, cm^2^/(kg/m^2^) *^2^*	0.128	0.013	<0.001	0.093	0.011	<0.001	−0.196	0.139	0.159
SMI_CT_, cm^2^/(kg/m^2^) *^3^*	0.062	0.012	<0.001	0.045	0.010	<0.001	−0.108	0.146	0.457
Female									
SMI_CT_, cm^2^/(kg/m^2^) *^1^*	0.067	0.016	<0.001	0.059	0.013	<0.001	0.109	0.231	0.637
SMI_CT_, cm^2^/(kg/m^2^) *^2^*	0.066	0.016	<0.001	0.060	0.013	<0.001	0.157	0.232	0.499
SMI_CT_, cm^2^/(kg/m^2^) *^3^*	0.028	0.015	0.059	0.032	0.012	0.010	0.272	0.239	0.256

*^1^* Adjusted for age and body mass index (BMI). *^2^* Adjusted for age, BMI, smoking, and exercise. *^3^* Adjusted for age, BMI, smoking, exercise, systolic blood pressure (BP), diastolic BP, height, total cholesterol, triglycerides, high-density lipoprotein (HDL) cholesterol and hemoglobin A1c. β linear regression coefficient; SE, standard error of β.

**Table 4:** Multiple linear regression analysis of lung function according to CT-measured skeletal muscle index (SMI_CT_) quartiles.

Variable	Q1	Q2	Q3	Q4	*p* for Trend
Male					
FVC, liters					
Model 1	Reference	0.18 (0.10, 0.26)	0.29 (0.21, 0.38)	0.40 (0.31, 0.49)	<0.001
Model 2	Reference	0.18 (0.10, 0.26)	0.28 (0.20, 0.37)	0.39 (0.30, 0.48)	<0.001
Model 3	Reference	0.09 (0.01, 0.16)	0.14 (0.06, 0.21)	0.18 (0.10, 0.26)	<0.001
FEV1, liters					
Model 1	Reference	0.11 (0.04, 0.18)	0.19 (0.12, 0.25)	0.28 (0.20, 0.35)	<0.001
Model 2	Reference	0.10 (0.04, 0.17)	0.18 (0.11, 0.25)	0.28 (0.20, 0.35)	<0.001
Model 3	Reference	0.04 (−0.02, 0.10)	0.08 (0.01, 0.14)	0.12 (0.05, 0.19)	0.007
Female					
FVC, liters					
Model 1	Reference	0.10 (−0.00, 0.20)	0.17 (0.07, 0.27)	0.23 (0.12, 0.34)	0.001
Model 2	Reference	0.09 (−0.01, 0.19)	0.17 (0.06, 0.27)	0.23 (0.11, 0.34)	0.001
Model 3	Reference	0.08 (−0.01, 0.17)	0.12 (0.02, 0.21)	0.14 (0.03, 0.24)	0.047
FEV1, liters					
Model 1	Reference	0.05 (−0.03, 0.13)	0.10 (0.02, 0.19)	0.18 (0.09, 0.27)	0.001
Model 2	Reference	0.04 (−0.04, 0.13)	0.11 (0.02, 0.19)	0.19 (0.09, 0.28)	0.001
Model 3	Reference	0.03 (−0.05, 0.11)	0.07 (−0.01, 0.15)	0.12 (0.03, 0.21)	0.056

β regression coefficient (95% confidence interval). 1 Adjusted for age and body mass index (BMI). 2 Adjusted for age, BMI, smoking, and exercise. 3 Adjusted for age, BMI, smoking, exercise, systolic blood pressure (BP), diastolic BP, height, total cholesterol, triglyceride, high-density lipoprotein (HDL) cholesterol and hemoglobin A1c. Cutoff values of SMI_CT_ quartiles (cm^2^/(kg/m^2^)) for males, 8.53, 9.29, 10.15; and for females, 7.26, 7.94, 8.94.

**Table 5:** Multiple logistic regression analysis of subjects with low PFT values according to CT-measured skeletal muscle index (SMI_CT_) quartiles.

Variable	Q4	Q3	Q2	Q1	***p* for trend**
Total					
Predicted FVC (%) <80% *^1^*	1.00 (reference)	1.94 (1.00, 3.79)	2.00 (0.98, 4.05)	3.52 (1.75, 7.10)	0.004
Predicted FEV1 (%) <80% *^1^*	1.00 (reference)	1.84 (0.77, 4.37)	2.18 (0.91, 5.23)	2.55 (1.03, 6.31)	0.192
Male					
Predicted FVC (%) <80% *^2^*	1.00 (reference)	2.02 (0.97, 4.22)	1.89 (0.87, 4.10)	3.09 (1.43, 6.70)	0.030
Predicted FEV1 (%) <80% *^2^*	1.00 (reference)	2.10 (0.75, 5.85)	2.58 (0.94, 7.04)	2.62 (0.92, 7.51)	0.225
Female					
Predicted FVC (%) <80% *^2^*	1.00 (reference)	1.94 (0.34, 11.02)	3.58 (0.55, 23.14)	9.21 (1.50, 56.44)	0.094
Predicted FEV1 (%) <80% *^2^*	1.00 (reference)	1.90 (0.29, 12.30)	1.55 (0.10, 23.09)	3.08 (0.29, 32.48)	0.807

Odds ratios (95% confidence interval). *^1^* Adjusted for age, sex, body mass index (BMI), smoking, exercise, systolic blood pressure (BP), diastolic BP, height, total cholesterol, triglyceride, high-density lipoprotein (HDL) cholesterol and hemoglobin A1c. *^2^* Adjusted for age, BMI, smoking, exercise, systolic BP, diastolic BP, height, total cholesterol, triglyceride, HDL cholesterol and hemoglobin A1c. Cutoff values of SMI_CT_ quartiles (cm^2^/(kg/m^2^)) for males, 8.53, 9.29, 10.15; and for females, 7.26, 7.94, 8.94.

## Supplementary Materials

The following are available online at https://www.mdpi.com/2077-0383/8/5/667/s1, Table S1: Comparison of the baseline characteristics of males according to CT-measured skeletal muscle index (SMICT) quartiles, Table S2: Comparison of the baseline characteristics of females according to CT-measured skeletal muscle index (SMICT) quartiles, Table S3: Comparison of PFT values between males according to CT-measured skeletal muscle index (SMICT) quartiles, Table S4: Comparison of PFT values between females according to CT-measured skeletal muscle index (SMICT) quartiles.
