# Supplementary material for: Association between CT-Measured Abdominal Skeletal Muscle Mass and Pulmonary Function
Source: J Clin Med. 2019 May 12;8(5):667. doi: 10.3390/jcm8050667 (PMC6572332; doi:10.3390/jcm8050667)
Supplement: Supplementary file 1 [file jcm-08-00667-s001.pdf]

Table S1. Comparison of the baseline characteristics of males according to CT-measured skeletal muscle index (SMI_{CT}) quartiles.

Variable	Total (n=1406)	SMI_{CT} quartiles				p for trend
		Q1 (n=352)	Q2 (n=351)	Q3 (n=351)	Q4 (n=352)	
Age, years	53.5 ± 9.2	55.9 ± 9.0	53.7 ± 9.1	52.6 ± 8.4	51.6 ± 9.6	<0.001
Systolic BP, mmHg	119.2 ± 12.9	121.6 ± 12.1	120.8 ± 12.7	117.8 ± 13.4	116.7 ± 12.8	<0.001
Diastolic BP, mmHg	78.5 ± 10.1	79.5 ± 9.9	80.3 ± 9.8	77.4 ± 10.3	76.9 ± 9.8	<0.001
Height, cm	170.2 ± 5.6	167.7 ± 5.4	169.8 ± 5.2	171.0 ± 5.2	172.3 ± 5.5	<0.001
Weight, kg	71.1 ± 8.7	72.4 ± 8.7	72.1 ± 8.9	71.0 ± 8.0	68.7 ± 8.7	<0.001
BMI, kg/m^2	24.5 ± 2.5	25.7 ± 2.5	25.0 ± 2.3	24.3 ± 2.2	23.1 ± 2.3	<0.001
WC, cm	88.0 ± 6.8	91.0 ± 6.5	89.1 ± 6.6	87.6 ± 5.8	84.2 ± 6.6	<0.001
Smoking						
Never	314 (22.3%)	94 (26.7%)	84 (23.9%)	64 (18.2%)	72 (20.5%)	0.014
Ex-smoker	752 (53.5%)	196 (55.7%)	194 (55.3%)	186 (53.0%)	176 (50.0%)	0.104
Current smoker	340 (24.2%)	62 (17.6%)	73 (20.8%)	101 (28.8%)	104 (29.5%)	<0.001
Exercise						
No regular exercise	364 (25.9%)	107 (30.4%)	88 (25.1%)	78 (22.2%)	91 (25.9%)	0.115
Moderate activity	505 (35.9%)	103 (29.3%)	124 (35.3%)	150 (42.7%)	128 (36.4%)	0.012
Rigorous activity (≥3/week)	537 (38.2%)	142 (40.3%)	139 (39.6%)	123 (35.0%)	133 (37.8%)	0.291
Muscle, cm^2	229.2 ± 29.4	204.2 ± 22.1	222.8 ± 20.8	234.9 ± 21.4	254.9 ± 27.0	<0.001
$SMI_{CT,}$ $cm^2/(kg/m^2)$	9.4 ± 1.3	8.0 ± 0.5	8.9 ± 0.2	9.7 ± 0.3	11.1 ± 0.9	<0.001
TC, mg/dL	189.9 ± 33.3	192.9 ± 34.0	192.9 ± 36.3	187.5 ± 32.3	186.4 ± 29.9	0.006
TG, mg/dL	131.5 ± 82.2	142.0 ± 90.8	141.3 ± 89.0	130.6 ± 80.1	112.2 ± 62.7	<0.001
HDL cholesterol, mg/dL	50.3 ± 11.5	49.7 ± 11.4	49.3 ± 10.7	49.3 ± 11.0	53.0 ± 12.4	<0.001
LDL cholesterol, mg/dL	119.6 ± 29.1	123.8 ± 30.2	120.0 ± 29.0	118.6 ± 30.7	116.5 ± 26.2	0.022
Fasting glucose, mg/dL	101.0 ± 17.3	101.7 ± 16.3	102.1 ± 16.5	100.5 ± 17.1	99.6 ± 18.9	0.053

HbA1c, %	5.8 ± 0.5	5.8 ± 0.5	5.8 ± 0.5	5.7 ± 0.6	5.7 ± 0.6	0.017

Data are presented as the mean ± standard deviation or as a percentage (%).

Cutoff values of SMI_{CT} quartiles ($cm^2/(kg/m^2)$) for males, 8.53, 9.29, 10.15. Q: quartile; BP: blood pressure; BMI: body mass index; WC: waist circumference; TC: total cholesterol; TG: triglyceride; LDL: low-density lipoprotein; HDL: high-density lipoprotein; HbA1c: hemoglobin A1c.

Table S2. Comparison of the baseline characteristics of females according to CT-measured skeletal muscle index (SMI_{CT}) quartiles.

Variable	Total (n=501)	SMI_{CT} quartiles Q1 (n=126)	Q2 (n=125)	Q3 (n=125)	Q4 (n=125)	p for trend
Age, years	52.2 ± 9.2	55.1 ± 8.5	52.9 ± 9.0	51.3 ± 8.9	49.7 ± 9.4	<0.001
Systolic BP, mmHg	111.8 ± 14.4	115.1 ± 15.1	112.6 ± 13.6	112.0 ± 13.6	107.5 ± 14.3	<0.001
Diastolic BP, mmHg	69.8 ± 10.2	71.5 ± 9.6	70.5 ± 10.5	70.0 ± 10.4	67.4 ± 10.2	0.001
Height, cm	158.7 ± 5.0	157.3 ± 5.7	158.1 ± 5.2	159.2 ± 4.0	160.4 ± 4.5	<0.001
Weight, kg	54.8 ± 6.8	58.3 ± 7.6	55.8 ± 6.5	53.8 ± 5.7	51.2 ± 5.1	<0.001
BMI, kg/m^2	21.7 ± 2.5	23.6 ± 2.7	22.3 ± 2.0	21.2 ± 2.0	19.9 ± 1.7	<0.001
WC, cm	79.3 ± 6.8	83.7 ± 6.4	81.2 ± 6.2	78.1 ± 5.5	74.0 ± 5.1	<0.001
Smoking						
Never	465 (92.8%)	122 (96.8%)	118 (94.4%)	115 (92.0%)	110 (88.0%)	0.005
Ex-smoker	17 (3.4%)	1 (0.8%)	5 (4.0%)	5 (4.0%)	6 (4.8%)	0.096
Current smoker	19 (3.8%)	3 (2.4%)	2 (1.6%)	5 (4.0%)	9 (7.2%)	0.027
Exercise						
No regular exercise	184 (36.7%)	50 (39.7%)	47 (37.6%)	46 (36.8%)	41 (32.8%)	0.265
Moderate activity	149 (29.7%)	43 (34.1%)	32 (25.6%)	38 (30.4%)	36 (28.8%)	0.538

Rigorous activity (≥3/week)	168 (33.5%)	33 (26.2%)	46 (36.8%)	41 (32.8%)	48 (38.4%)	0.083
Muscle, cm^2	176.0 ± 23.2	156.8 ± 18.2	170.4 ± 15.7	178.2 ± 16.9	198.9 ± 19.0	<0.001
$SMI_{CT,}$ $cm^2/(kg/m^2)$	8.2 ± 1.4	6.7 ± 0.5	7.6 ± 0.2	8.4 ± 0.3	10.0 ± 1.0	<0.001
TC, mg/dL	198.3 ± 34.9	203.7 ± 36.9	204.8 ± 38.7	191.7 ± 29.2	193.0 ± 32.1	0.015
TG, mg/dL	93.7 ± 60.3	105.1 ± 45.0	113.4 ± 86.3	84.5 ± 48.2	72.1 ± 42.5	<0.001
HDL cholesterol, mg/dL	60.2 ± 13.3	55.9 ± 11.9	59.4 ± 13.3	61.6 ± 13.1	64.1 ± 13.7	<0.001
LDL cholesterol, mg/dL	120.0 ± 30.5	128.4 ± 31.9	124.6 ± 35.1	113.5 ± 25.2	114.3 ± 27.5	0.008
Fasting glucose, mg/dL	92.7 ± 12.7	96.2 ± 11.9	93.9 ± 12.4	92.3 ± 16.1	88.3 ± 7.7	<0.001
HbA1c, %	5.7 ± 0.4	5.8 ± 0.4	5.8 ± 0.5	5.7 ± 0.5	5.6 ± 0.3	<0.001

Data are presented as the mean ± standard deviation or as a percentage (%).

Cutoff values of SMI_{CT} quartiles ($cm^2/(kg/m^2)$) for females, 7.26, 7.94, 8.94. Q: quartile; BP: blood pressure; BMI: body mass index; WC: waist circumference; TC: total cholesterol; TG: triglyceride; LDL: low-density lipoprotein; HDL: high-density lipoprotein; HbA1c: hemoglobin A1c.

Table S3. Comparison of PFT values between males according to CT-measured skeletal muscle index (SMI_{CT}) quartiles.

Variable	Total (n=1406)	SMI_{CT} quartiles				p for trend
		Q1 (n=352)	Q2 (n=351)	Q3 (n=351)	Q4 (n=352)	
FVC, liters	4.2 ± 0.6	3.9 ± 0.6	4.1 ± 0.6	4.3 ± 0.6	4.4 ± 0.6	<0.001
FVC, % predicted	96.2 ± 11.3	94.0 ± 11.0	96.0 ± 11.4	97.0 ± 11.4	98.0 ± 11.0	<0.001
Predicted FVC (%) < 80%	86 (6.1%)	30 (8.5%)	20 (5.7%)	22 (6.3%)	14 (4.0%)	0.022
FEV1, liters	3.3 ± 0.6	3.1 ± 0.5	3.3 ± 0.5	3.4 ± 0.6	3.5 ± 0.6	<0.001
FEV1, % predicted	103.3 ± 13.5	101.4 ± 13.7	102.7 ± 13.5	103.8 ± 13.8	105.3 ± 12.6	<0.001
Predicted FEV1 (%) < 80%	54 (3.8%)	19 (5.4%)	17 (4.8%)	12 (3.4%)	6 (1.7%)	0.006
FEV1/FVC (%)	78.9 ± 6.4	78.8 ± 6.4	78.6 ± 6.1	78.8 ± 6.4	79.3 ± 6.5	0.153
FEV1/FVC < 70%	95 (6.8%)	26 (7.4%)	23 (6.6%)	24 (6.8%)	22 (6.2%)	0.601

Data are presented as the mean ± standard deviation or as a percentage (%). Cutoff values of SMI_{CT} quartiles ($cm^2/(kg/m^2)$) for males, 8.53, 9.29, 10.15.

Table S4. Comparison of PFT values between females according to CT-measured skeletal muscle index (SMI_{CT}) quartiles.

Variable	Total (n=501)	SMI_{CT} quartiles				p for trend
		Q1 (n=126)	Q2 (n=125)	Q3 (n=125)	Q4 (n=125)	
FVC, liters	3.0 ± 0.4	2.9 ± 0.5	3.0 ± 0.4	3.1 ± 0.4	3.1 ± 0.4	<0.001
FVC, % predicted	98.2 ± 12.0	98.0 ± 11.8	98.8 ± 10.9	98.3 ± 13.3	97.6 ± 11.9	0.539
Predicted FVC (%) < 80%	15 (3.0%)	5 (4.0%)	4 (3.2%)	3 (2.4%)	3 (2.4%)	0.418
FEV1, liters	2.5 ± 0.4	2.3 ± 0.4	2.4 ± 0.4	2.5 ± 0.4	2.6 ± 0.4	<0.001
FEV1, % predicted	106.7 ± 14.0	107.3 ± 13.9	106.7 ± 12.9	106.2 ± 15.2	106.8 ± 13.9	0.952
Predicted FEV1 (%) < 80%	10 (2.0%)	2 (1.6%)	2 (1.6%)	3 (2.4%)	3 (2.4%)	0.562
FEV1/FVC (%)	81.8 ± 6.2	81.5 ± 5.7	81.1 ± 5.6	81.6 ± 6.3	83.1 ± 7.1	0.008
FEV1/FVC < 70%	10 (2.0%)	2 (1.6%)	1 (0.8%)	4 (3.2%)	3 (2.4%)	0.387

Data are presented as the mean ± standard deviation or as a percentage (%). Cutoff values of SMI_{CT} quartiles ($cm^2/(kg/m^2)$) for females, 7.26, 7.94, 8.94.
